# Unusual Case of Life-Threatening Gastro Intestinal Bleed from a Splenic Artery Pseudoaneurysm: Case Report and Review of Literature

**DOI:** 10.1155/2019/8528906

**Published:** 2019-02-06

**Authors:** Puneet Menaria, Venkata Muddana

**Affiliations:** ^1^Department of Hospital Medicine, Aurora St. Luke's Medical Center, Suite 305, Medical Office Building 2, West K.K. River Parkway, Milwaukee, WI 53215, USA; ^2^Clinical Assistant Professor, Department of Gastroenterology, Aurora St. Luke's Medical Center, 2900 W. Oklahoma Avenue, Milwaukee, WI 53215, USA

## Abstract

Large upper gastro intestinal (GI) bleeding can be life-threatening. Splenic artery pseudoaenurysm (SAP) is rare but can cause massive upper GI bleeding. We report a case of a 57-year-old woman who had massive upper GI bleeding from SAP eroding into distal duodenum. Literature review shows SAP can bleed into stomach or pancreatic pseudocyst or biliary tree and peritoneal cavity; however, there are no previous reported cases of SAP bleeding into distal duodenum. Splenic artery embolization (SAE) is the preferred treatment for a bleeding SAP. Splenic infarcts can result following a SAE.

## 1. Background

Massive upper GI bleeds (UGIB) can be life-threatening. Common causes for these large bleeds include peptic ulcer disease, esophageal or gastric varices, mucosal tears of esophagus or fundus (Mallory-Weiss tear), erosive gastritis, erosive esophagitis, and Dieulafoy lesions.

Splenic artery pseduoaneurysm (SAP) is rare [1].

Hemorrhage from SAP can present as massive UGIB either from bleeding into pancreatic duct (hemosuccus pancreaticus) or by eroding into the gastric wall [2–4]. Pancreatitis and trauma are amongst the most common causes for SAP.

Untreated ruptured SAP has high mortality in excess of 90% [5]. Familiarity with this rare pathology is essential for timely diagnosis and intervention.

The case reported is a rare instance of SAP eroding into fourth part of duodenum, resulting in a massive UGIB. To our knowledge this is the first reported case of SAP eroding into distal duodenum.

## 2. Case Report

### 2.1. Initial Presentation

A 57-year-old female presented to emergency room with left lower quadrant abdominal pain. She was afebrile with vital signs within normal limits. WBC count, liver function studies, and lipase were within normal limits.

She reported several episodes of abdominal pain in last 3 years related to recurrent diverticultis. She also reported a remote history of gastric ulcer. She specifically denied any previous history of pancreatitis. She reported a family history of pancreatic cancer (mother in her 80's). She denied any prior h/o trauma. She drank alcohol occasionally and had a 30 pack year history of cigarette smoking.

**Figure 1:**
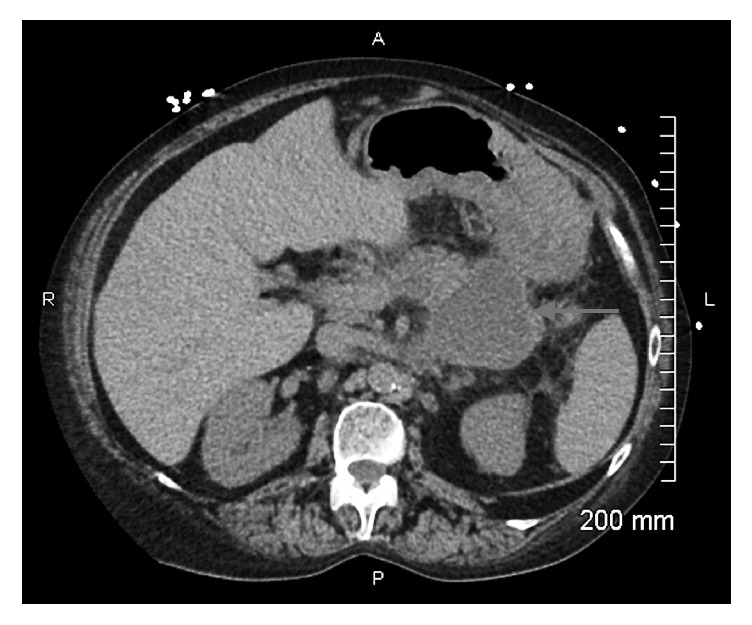
CT image; pancreatic tail lesion.

**Figure 2:**
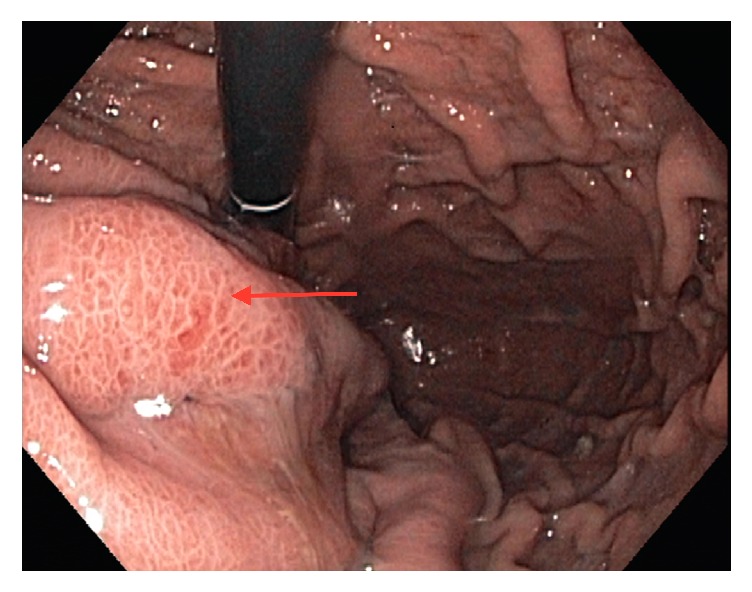
Endoscopy image; extrinsic bulge with erythematous mucosa.

Due to her history of diverticulitis a CT scan of abdomen and pelvis was obtained which revealed a 6.9 x 6.1 cm cystic mass in the tail of the pancreas (Figure 1). Tumor markers (CEA and CA 19-9) were within normal range. Subsequently upper GI endoscopy (EGD) with endoscopic ultrasound (EUS) was performed. EGD showed an extrinsic bulge in the gastric fundus presumably secondary to pancreatic tail lesion. There was a small central ulceration and erythematous surrounding mucosa (Figure 2).

**Figure 3:**
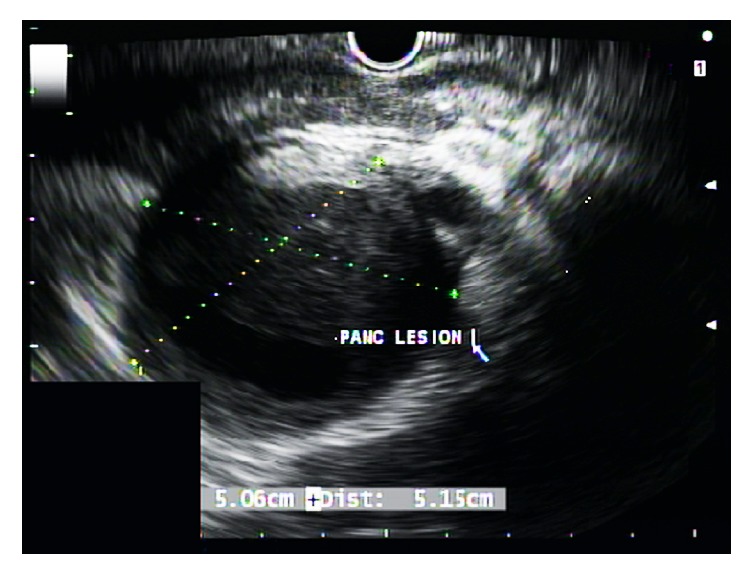
EUS image; pancreatic tail lesion with anechoic component.

On EUS, a hypoechoic 5 x 5.15 cm lesion with internal anechoic component (Figure 3) was seen in the tail of the pancreas. Using color flow Doppler Fine Needle Aspiration (FNA) was obtained.

Cytology showed no malignant cells and inflamed cyst contents were seen. Cyst fluid showed amylase 8433 and lipase 87352, with low CEA 2.2.

She was discharged home with a plan to repeat EUS with FNA in 3 months.

Prior to repeat EUS she presented to emergency room (ER) with massive hematemesis.

### 2.2. ER Course

She presented to the emergency room vomiting large volume of blood. She was hypotensive, diaphoretic, and appeared pale. She was hypotensive and tachycardic with hemoglobin of 6.4, presenting vitals and labs are summarized in Tables 1 and 2.

Despite aggressive fluid resuscitated profound hypotension persisted and vasopressors were commenced. She suffered pulseless electrical activity cardiac arrest; advanced cardiac life support (ACLS) was commenced; she had return of spontaneous circulation after 2 round of standard ACLS protocol. Massive transfusion protocol was initiated; she was transfused 4 units of O negative red blood cells and was continued on vasopressors.

With the previous endoscopy done 3 months ago showing gastric ulcer there was a very high clinical suspicion for an upper GI bleed related to gastric ulcer. Emergent upper GI endoscopy within hour of cardiovascular collapse showed stomach full of clotted blood limiting mucosal visualization. No clear lesion was identified. Immediate surgical consult within hour of EGD was obtained and patient was sent for emergent mesenteric angiogram.

**Figure 4:**
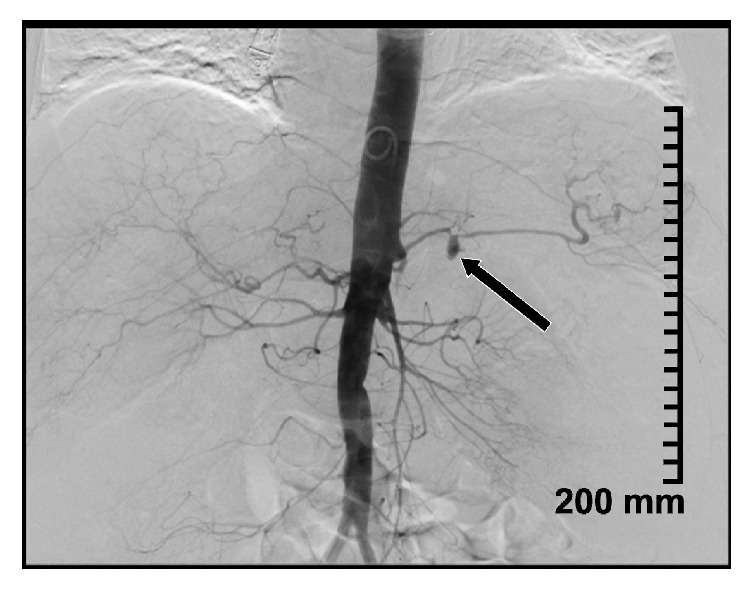
Bleeding splenic artery pseudoaneurysm seen on angiogram.

**Figure 5:**
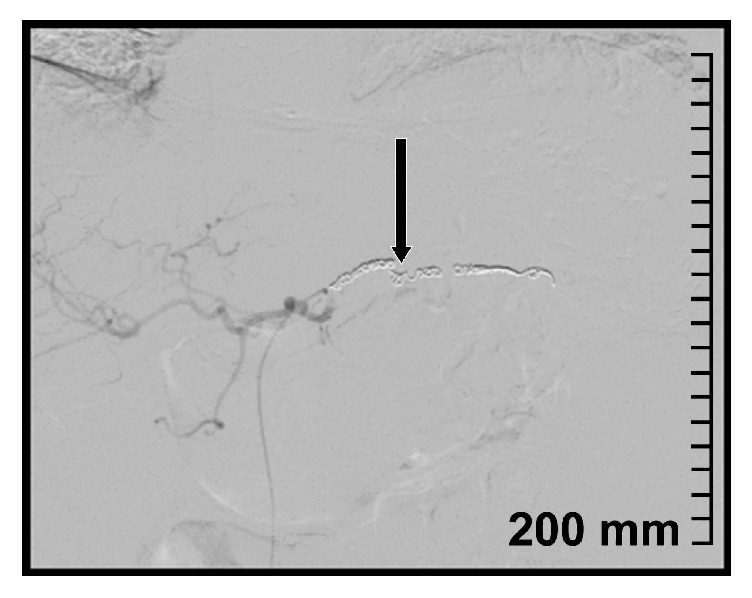
Post coil embolization angiogram with no bleeding.

Selective splenic artery angiogram confirmed a pseudoaneurysm with active hemorrhage (Figure 4). Coil embolization was performed with 6 mm x 7 cm; 3 mm x 7 cm (2), 3 mm x 5 cm (3) Nester ® coils (6 total); postembolization angiography demonstrated no leakage from SAP as well as no forward flow in the splenic artery (Figure 5).

**Figure 6:**
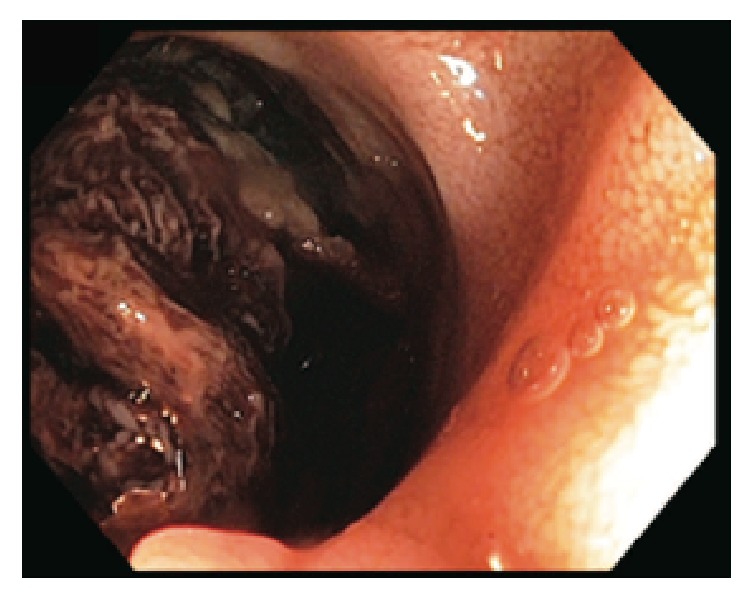
Endoscopy image; ulcer located in distal duodenum.

**Figure 7:**
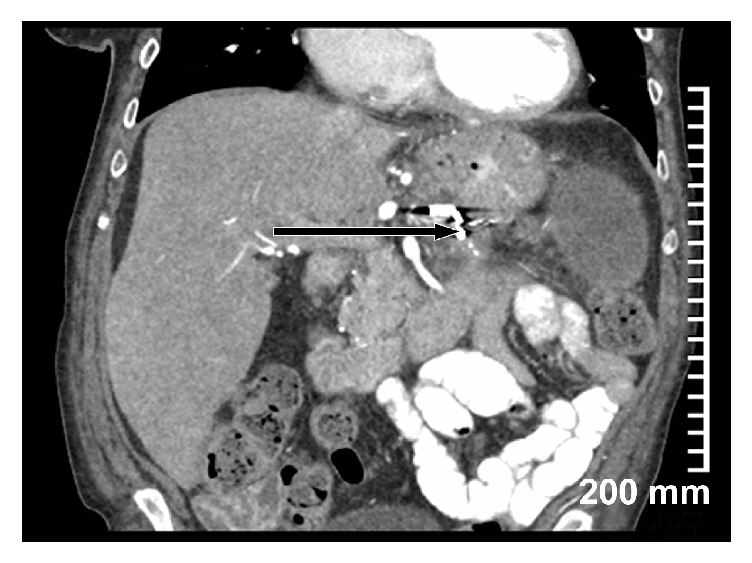
Coronal CT abdomen image showing vascular coils in close proximity of distal duodenum.

Repeat EGD was done 5 days later for ongoing drop in hemoglobin with persistent melena. EGD showed no abnormalities in the stomach; therefore the scope was advanced into deep duodenum where a large deep ulcer was seen in the fourth part of the duodenum (Figure 6). Multimodality imaging confirmed embolization coil to be in close proximity to the distal duodenum/proximal jejunum (Figure 7) further confirming the site of erosion of SAP in distal duodenum.

**Figure 8:**
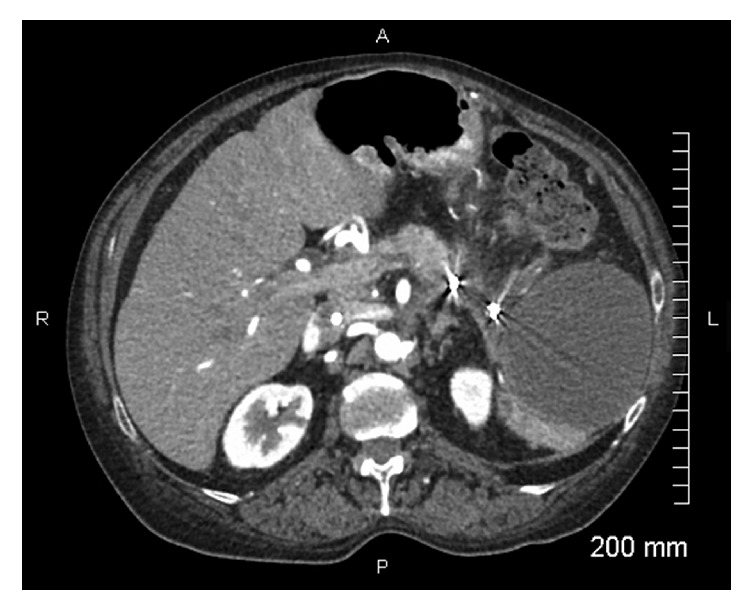
CT image: infarcted spleen.

Patient developed left upper quadrant abdominal pain and fever 48 hours after splenic artery embolization (SAE); splenic infarction was suspected. This was dealt with use of narcotics for analgesia and acetaminophen for fever; broad-spectrum antibiotic was administered for 1 week. Fever gradually resolved over next 3-4 days; however pain persisted for 2 weeks. Patient was discharged home in a stable condition after 14 days. Near complete splenic infarction was confirmed at 2 months on follow-up CT scan (Figure 8). No recurrent bleeding was noted at 2 and 6 months of follow-up.

## 3. Discussion

Pseudoaneurysms of visceral artery are rare; amongst these, pseudoaneurysm of splenic artery is even rarer. In fact in a large multicentre study for patients with diagnosis of visceral artery aneurysm only 10 cases were identified with SAP on review of 18 years of record. The same study identified only 157 reported cases of SAP [1]. A handful more cases of acute bleeding from SAP has been reported recently in English literature [6, 7].

Splenic artery, a branch of the celiac axis, is the major arterial branch for the pancreas and the spleen. Before reaching the spleen at the splenic hilum, the splenic artery runs along the superior border of the pancreas, giving branches, which traverses the body of the pancreas. Close vicinity to the pancreas makes the splenic artery vulnerable to enzymatic leaks from acute and chronic inflammation of the pancreas. Enzymatic leak is postulated to cause injury to the arterial wall of the splenic artery, resulting in true and pseudoaneurysm [8]. Pseudoaneurysms can also result from direct compression or ischemia from the pancreatic pseudocyst in addition to the vascular erosion from enzymes within the pseudocyst [9]. Other reported causes for SAP include abdominal trauma, peptic ulcer disease, iatrogenic from endovascular, or laparoscopic surgical explorations and idiopathic [1, 4, 10, 11].

While true aneurysms of splenic artery are often identified incidentally, SAP is almost always detected due to associated clinical symptoms [12]. In a review of the literature, only 2.5% of cases of SAP presented incidentally [1]. Hematochezia, hematemesis, and abdominal pain are common presenting symptoms of SAP; other symptoms reported are nausea, vomiting, flank pain, and abdominal mass [1],

While SAP is amongst rare causes for an UGIB, hemorrhage is reported to be the most common presentation of patients with SAP [1]. Tesiier et al. reported hemorrhage as the presenting symptom in 74/157 (47%) of patients with SAP; site of bleeding could be identified in 59 of 74 patients, including bleeding in the pancreatic duct 25 (42%), stomach 13 (22%), peritoneal cavity 11 (20%), and colon 9 (15%). No cases were identified to have bleeding in the duodenum like the case reported. Interestingly size of SAP does not predict the risk of rupture; smaller SAP are at similar risk of rupture [1]. A ruptured SAP has a mortality in excess of 90% [5]. UGI hemorrhage from SAP can have a relapsing course; endoscopies performed between episodes of active bleeding can have false negative results [2, 13].

Computed tomography (CT) scans are commonly used to establish the diagnosis of SAP. On CT, SAP has been identified in pseudocysts, appearing as focal regions of enhancement in the low-density intracystic fluid [14]. Peripancreatic pseudoaneurysms may show increased attenuation on unenhanced scans, and the perfused portion will enhance strongly after contrast infusion [15]. This feature of SAP mimics pancreatic islet cell tumor, which can similarly enhance on the arterial phase [16]. Though easily available and low cost, ultrasound studies have been reported to be rarely conclusive and have limitations of being operator-dependent [17, 18]. With either CT or ultrasound, SAP may be mistaken for pseudocysts or other peripancreatic fluid collections. High density within a peripancreatic fluid collection should raise the suspicion of clot within a pseudoaneurysm [1].

Magnetic resonance imaging (MRI) and magnetic resonance angiography have improved spatial resolution but do have limitations of availability; this modality cannot be utilized in patients with pacemakers or aneurysm clips, those suffering from claustrophobia, and those unable to hold their breath [19].

The most commonly used and most reliable way to diagnose SAP is catheter angiography. This technique also allows the benefit of transcatheter embolization in appropriate patients; thus all patients with hemodynamic instability with suspected SAP should undergo angiography if tolerable [1].

The diagnosis should be suspected in patients with pancreatitis who develop either upper gastrointestinal bleeding without an obvious cause or in whom a contrast-enhanced lesion is demonstrated within or adjacent to a suspected pseudocyst as determined by CT scan [20].

Rarity of SAP necessitates a high degree of suspicion for correct identification of the lesion.

SAP have been reported to have a high risk of spontaneous hemorrhage (37%), with the mortality rate approaching 90% when untreated [14, 21]. Size of SAP does not predict risk of rupture; therefore all SAP regardless of size shall undergo treatment.

Conventional treatment included open surgical technique and including repair of SAP and ligation of artery or splenectomy with partial pancreatectomy depending on location of SAP. Tossier et al. in a 2003 review identified surgical repair of SAP with splenectomy with or without distal pancreatectomy to be the most durable treatment; surgical intervention was more successful than endovascular embolization in patients with SAP related to pseudocyst formation [1].

With advancements in field of angiography and wider availability, endovascular procedure has increasingly become initial treatment of choice. In the last decade Jia et al. [22], Loffroy et al. [23], and Satoshi et al. [24] have reported endovascular management of SAA and SAP utilizing embolization techniques with use of (coils, glue or sponge) as well as endovascular stent and stent graft with efficacy ranging from 88-100%. The authors described a perioperative mortality of zero (0%). Surgery for splenic artery aneurysm has been reported with significant comorbidity (9%) and mortality of up to 1.9% [25].

Endovascular treatment allows for evaluation of collateral circulation and offers a viable option in patients who are high risk for surgery [22–24]. With endovascular techniques preservation of blood flow in splenic artery desirable however not essential as collateral flow it is expected to prevent splenic infarction [23]. Complications following endovascular procedures include postembolization syndrome (pain and fever), splenic infarction, splenic abscess, and rebleeding requiring surgical intervention [22–24].

*Splenic Infarction after Splenic Artery Embolization (SAE)*. Satoshi et al. [26] reported a 100% incidence (16/16 patients) of fever and pain after SAE even in patients with preserved blood flow. Splenic infarction of varying degree has been reported in up to 66 % of cases following SAE [22]. This complication is reported to be more common after embolization of distal splenic artery or hilar/intrasplenic aneurysms [12, 22, 26]. Stephen et al. [26] using contrast enhanced CT measured spleen volume pre and post SAE and identified a greater loss of spleen volume after distal SAE. A meta-analysis found similar results of increased splenic infarcts and splenic abscess following distal SAE [27]. Classification of Proximal/Distal SAE has slightly varied in different studies; however most studies considered splenic artery segment before bifurcation into splenic branches as proximal segment. Authors have described favorable outcome managing splenic infarctions with use of antibiotics, analgesics, and antipyretics [22, 24].

Immune function compromise following SAE has been a matter of investigation and much debate. Available evidence [26–30] suggests that spleen immune function is likely preserved after SAE; however, there is still no single test available to demonstrate this. A critical splenic mass required to preserve splenic function is not known. Standard postsplenectomy immunizations are not advised following a SAE procedure.

### 3.1. Conclusion

In summary, the most important factor in identifying ruptured SAP as the source of GI bleeding is considering the diagnosis. The diagnosis should be considered in patients with confirmed or suspected history of pancreatitis who develop upper gastrointestinal bleeding without an obvious cause or in whom a contrast-enhanced lesion is demonstrated on CT scan within or adjacent to a pancreatic pseudocyst.

Upper GI hemorrhage from SAP can happen in the distal duodenum. CT angiography is useful in establishing a diagnosis and commencement of appropriate treatment. Emergent IR angiography (digital subtraction angiography) in hemodynamically unstable patients is helpful both diagnostically and therapeutically. Splenic Infarctions are more common following a distal splenic artery embolization. Evidence suggests preservation of spleen immune function following splenic artery embolization.

## Figures and Tables

**Table 1:** 

ED Triage Vitals	

Temp	97.5°F (36.4°C)
Pulse	105
Resp	20
BP	86/55
SpO2	100%
Height	5′ 3′′ (1.6 m)
Weight	163 lb 2.3 oz (74 kg)

**Table 2:** 

Test	Measured	Normal Range	Test	Measured value	Normal Range

WBC	17.7	4.2 - 11.0 K/mcL	Total Bilirubin	0.4	0.2-1.0
RBC	2.16	4.00 - 5.20 mil/mcL	AST/SGOT	26	<38 units/L
HGB	6.4	12.0 - 15.5 g/dL	ALT/SGPT	12	<79 Units/L
HCT	19.4	36.0 - 46.5 %	Alkaline Phosphatase	126	45-117 units/L
MCV	89.8	78.0 - 100.0 fl	Total Protein	6.6	6.4-8.2 g/dl
MCH	29.6	26.0 - 34.0 pg	Albumin	2.9	3.6-5.1 g/dl
MCHC	33.0	32.0 - 36.5 g/dL	Globulin	3.7	2.0-4.0 g/dl
RDW-CV	16.0	11.0 - 15.0 %	A/G Ratio	0.8	1.0-2.4
PLT	323	140 - 450 K/mcL	Lipase	102	73-393 Units/L
